# “I didn’t Notice that You Were Watching Me”: Exploring a User Acceptance Study to Conduct Cultural Domain Analysis Online During the COVID-19 Pandemic

**DOI:** 10.1177/16094069231164602

**Published:** 2023-04-19

**Authors:** Caroline Ackley, Diego Garcia Rodriguez, Giovanni Villa

**Affiliations:** 1 Department of Global Health & Infection, 12190 Brighton & Sussex Medical School, University of Sussex, Brighton, UK; 2 School of Sociology and Social Policy, University of Nottingham, Nottingham, UK; 3 Department of Genitourinary Medicine & Infectious Diseases, St James’s Hospital, Dublin, Ireland

**Keywords:** cultural domain analysis, COVID-19, qualitative research, user acceptance study, online and digital research

## Abstract

This article explores the implementation process of a User Acceptance Study to evaluate the feasibility of conducting cultural domain analysis (CDA) online during the COVID-19 pandemic. We conducted 19 cultural domain analysis sessions involving three techniques: free listing, pile sorts, and rank ordering. A diverse set of participants were recruited to help assess requirements and needs that researching online involves. We found that conducting CDA online is a feasible research method that offers benefits such as generating large amounts of data, making participants feel comfortable joining sessions from a safe space, providing anonymity, reducing research costs such as time and travel, and eliciting large numbers of responses. We also identified several factors for consideration when implementing CDA online and provide recommendations for improvement, including the aesthetics of the digital software employed, user accessibility and digital literacy, participants’ environments, Internet connection, and online-specific ethical issues.

## Introduction

This article draws upon a User Acceptance Study (UAS) that was designed and implemented by a team of researchers at the Brighton and Sussex Medical School (BSMS) to evaluate the feasibility of implementing cultural domain analysis (CDA) online. The UAS was intended to gather evidence to test the feasibility of doing research online for a future study engaging with people living with HIV amid the COVID-19 pandemic (Villa et al., 2022). Prior to implementing the UAS, the research team developed a protocol for a qualitative study exploring the impact of HIV drug regimens on people living with HIV and receiving care in Brighton, United Kingdom (UK). As part of the process to obtain ethical approval from the Health Research Authority Ethics Committee, the team was asked to demonstrate the feasibility of conducting the research project online. This had to do with the developments of the COVID-19 pandemic, which led to the passing of legislation restricting the movement of citizens in the UK. Considering that such regulations could impede meeting participants physically as well as aiming to reduce any impact on their health by reducing in-person meetings, we adapted our methodology to online. This paper describes the UAS we conducted and provides guidance for future studies that employ CDA online.

We begin with a brief introduction to digital research and a review of the literature on digital research during the pandemic. Next, we describe CDA, followed by how CDA was implemented during this UAS. Then, we outline the challenges and benefits faced while conducting the UAS. Finally, drawing upon the participants’ and our own experiences, we conclude with a set of lessons learnt and recommendations.

## Doing Digital Research During a Pandemic

The UAS was conducted due to the need to quickly test the acceptability and validity of switching CDA from in-person to online, thus making this a digital study. Digital methods have been described through terms such as ‘virtual’, ‘Internet’ or ‘online’ methods (Gubrium & Harper, 2012). And for many, digital environments are sometimes described in opposition to the ‘real’ world and depicted as alternative realities where actions are not as tangible as those taking place physically. As Tom Boellstorff argues, paraphrasing the work of Annette Markham, “a phrase like ‘in real life’ often demarcates those experiences that occur offline” (Boellstorff, 2015, p. 20). Therefore, the equation of ‘real’ with ‘offline’ raises an important inquiry regarding what doing research online means against in-person methods. In our UAS, this implied conducting research through a range of digital platforms, but also involved a mediated interaction that differed from more familiar, in-person approaches.

Digital methods are diverse and, as is the case with in-person research, employing digital methods requires a comprehension of the method in question and its appropriateness for the context where it is to be applied (Hughes et al., 2015, p. 154). Digital methods are not always a completely new set of methods, but often the adaptation of traditional ones (e.g., interviews, focus group discussions) to an online environment. Existing literature has explored, among other methods, online interviews (Ayling & Mewse, 2009; Bauman, 2015; Salmon, 2014), online surveys (Comley, 2002), and online focus group discussions (FGDs) (Colom, 2021; Reid & Reid, 2005; Woodyatt et al., 2016; Reisner et al., 2018). Scholarship has established an ontological distinction between the natively digital methods (i.e., specifically created for a new digital medium) and the digitised (i.e., those that have migrated to it) (Rogers, 2009, p. 5). Recent scholarship has explored lessons learnt to quickly adapt interviews and FGDs to the online sphere during the pandemic. The literature shows that the benefits of online research include participants being more comfortable, feeling safer and more engaged (Dodds & Hess, 2020), and the importance of preparation by reducing the number of participants, holding test calls, and preparing online user guides (Garcia et al., 2020). Additional qualitative methods have been adapted to online including rapid ethnographies (Collins et al., 2022), chat-based research (Luk et al., 2022), and photovoice (Badanta et al., 2021).

Despite the benefits of conducting online research, there are limitations including “the lack of non-verbal communication, poor set-up, and privacy and access issues” (Dodds & Hess, 2020, p. 203). Reflecting on the impact of COVID-19 on doing research with marginalised communities, Sevelius et al. (2020) identified three key challenges: (1) technological barriers for communities who lack access to the devices required or do not have the skills needed; (2) providing financial support to participants in exchange for their participation, and (3) preserving the safety of participants because of the absence of facilities where they could adhere to COVID-19 safety recommendations.

This UAS extends our knowledge on conducting CDA and research during the COVID-19 pandemic by offering some of the first insights into adapting CDA from in-person to online. Despite similar benefits and challenges to the other methods detailed above, conducting CDA and doing so online comes with unique insights which we discuss below.

## Cultural Domain Analysis

The heterogenous set of methods known as cultural domain analysis (CDA) first became popular in the 1960s, at the time of what some scholars call the “cognitive revolution” (Hjørland & Albrechtsen, 1995, p. 405). CDA can be defined as “an approach derived from cognitive anthropology to describe the contents, structure, and distribution of knowledge in organized spheres of experience, or cultural domains” (Gravlee et al., 2018, p. 165). As Borgatti explains, cultural domains can be understood as “categories” (including, for example, vegetables, illnesses, or animals), which are “about perceptions rather than preferences” (1998, p. 1) and shared and agreed among people. Multiple techniques can be used within the umbrella of CDA including, those we use, free listing, pile sorting, and rank ordering, as well as true-false/yes-no and sentence frame techniques, triad tests, and paired comparisons. CDA differs from other interview techniques, surveys, and FGDs in that it focuses on ‘things external to the people we interview and how those things are related to each other in people’s minds’ (Bernard, 2006, p. 301). CDA involves building folk taxonomies from data that participants provide on ‘what goes with what’ (ibid.).

While much literature on digital methods has paid attention to interviews, surveys and FGDs, little has been written about conducting CDA online. One of the few articles exploring CDA analysed the differences between pile sort data collected online versus face-to-face interviews and found that web-based collection of data mostly produces results that are comparable to in-person approaches (Gravlee et al., 2018). As they explain, there is “no evidence that collecting data online changes our understanding of the semantic structure in a consensual cultural domain” (ibid, p. 172). Employing the CDA technique pile sorting, Ford (2013) has highlighted benefits of going digital. Among them, she mentions the reduction of time and energy required to enter data, and the possibility to engage with more participants since they can join the sessions from the comfort of their home (Ford, 2013, p. 260). However, Ford also notes disadvantages related to (a) the potential loss of data when participants are not asked to “think aloud”, (b) the difficulties to revisit the different cards online when there are too many, and (c) potential software functionalities that may reduce the number of cards that participants create (ibid, p. 260).

## Implementing Cultural Domain Analysis in this User Acceptance Study

The UAS did not require ethical approval because the aim was to test the feasibility of conducting CDA online. However, ethical considerations were at the core of our research practice and discussed with the Research Governance and Ethics Committee (RGEC), who gave us written approval.

Prior to designing our methods and implementing them, we contacted the Technology Enhanced Learning (TEL) department at the University of Sussex. In addition to sharing their Accessibility Checklist (TEL, 2020), they provided recommendations on the software best suited to conducting CDA online, namely Padlet®. Padlet® provides ‘an online notice board’ where participants can ‘post notes on a common page’ (PDST, 2022). These can include ‘links, videos, images and document files,’ however, we used text only. Considering the benefits presented by Padlet®, we decided to choose it as the platform where online participants would free list, pile sort, and rank order their answers.

We recruited 19 participants from a diverse sample including different age groups (from 20 to 76 years old), genders (7 men, 12 women), social classes, locations (US and UK), and one participant with vision impairment. Participants also had caring responsibilities and differing levels of digital literacy and included social scientists, clinicians, patient representatives, and individuals who were not familiar with academic research. Participants took part in one CDA session each and provided feedback and suggestions for improvement.

**Figure 1.**
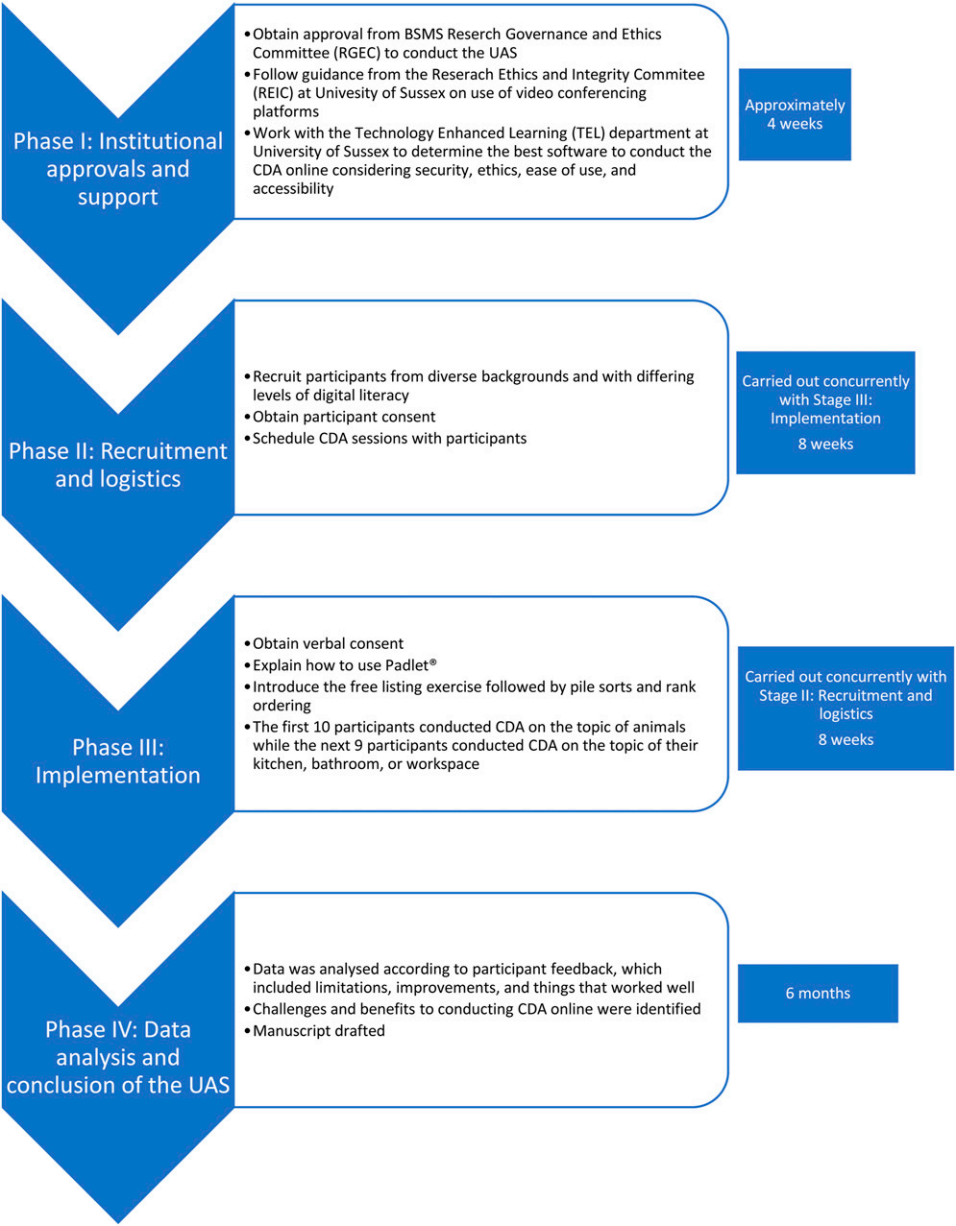
The phases of the UAS and timeline.

In what follows we describe the process of implementation of our UAS, including the three main study phases, which is summarised in Figure 1 below.

### Phase 1: Institutional Approvals and Support

Once the UAS plan was shared with BSMS RGEC and TEL, the team started designing the CDA topic guides. We followed the University of Sussex guidance published by the Research Ethics and Integrity Committee (REIC) on the use of video conferencing platforms in research (April, 2020). The REIC suggests researchers use Microsoft Teams®, Microsoft Skype for Business®, or Zoom for Education® for video conferencing due to both university compliance and the security provided by such platforms. We obtained advice on security, ethics, ease of use, and accessibility from BSMS REIC and TEL, and components of the study like text size and colour were considered for improved accessibility.

### Phase 2: Recruitment and Logistics

A member of our department acted as the Research Assistant and helped develop and send invitation emails to participants. This included a digital consent form and a participant information sheet. Reflecting on the recruitment process, the research assistant considered that, “in hindsight, offering one date and various times in the first email might have been better, as this meant waiting for participants to email back, before I could offer another day and time to the next participant” (field note, July 2020). Signed consent forms were saved in an encrypted university hard drive where participants were assigned numbers for anonymity.

### Phase 3: Implementation

After considering the variety of CDA techniques, our team chose free lists, pile sorts, and rank ordering. The first two CDA interviews were conducted with two research team members present instead of just one. This was for the practical reason that one of the researchers had never conducted CDA, as well as to identify any immediate challenges to conducting CDA online, and to become familiar with carrying out the methods online versus in-person. Once the researcher became comfortable using CDA and initial challenges were addressed the CDA sessions were conducted individually. The sessions started by obtaining verbal consent from the participants and providing a reminder of the objectives. Following this, the researchers explained how to use Padlet®, and introduced the free listing exercise followed by pile sorts and rank ordering. After spending about 5 minutes testing the features of Padlet®, it took the respondents approximately 5 to 10 minutes to complete each technique.

**Figure 2.**
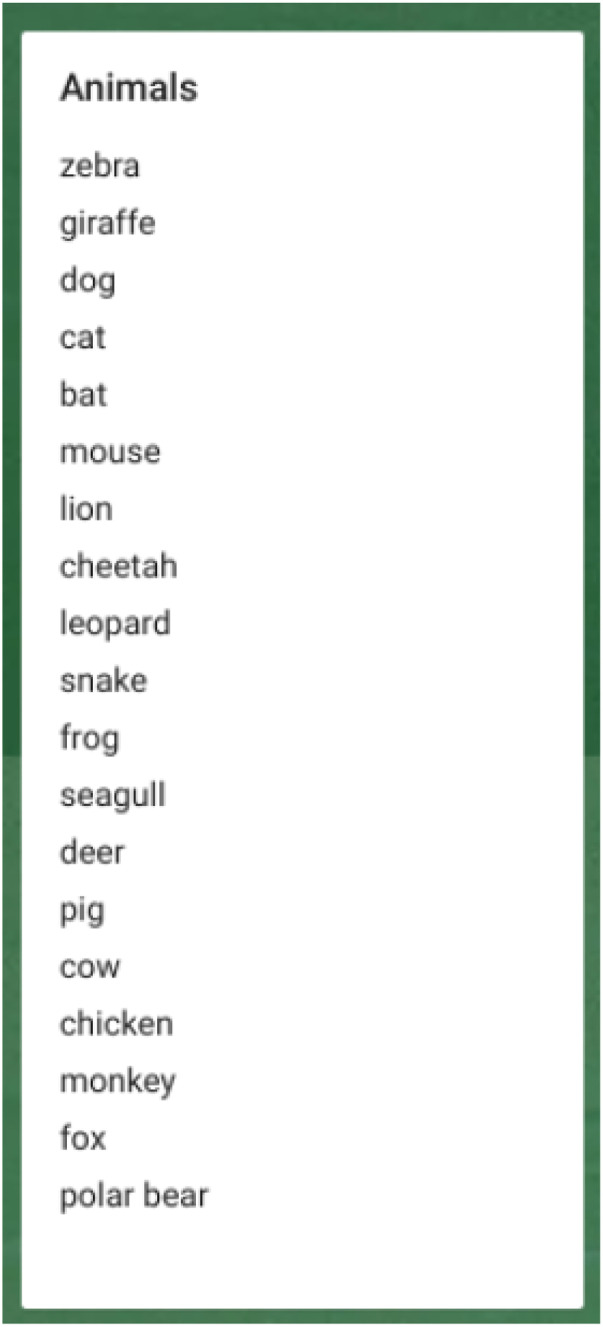
Example of free listing on Padlet®.

Looking to elicit responses on domain membership, we used free listing to ask participants to list all the elements they could think of within a domain. Free listing is a “simple, open-ended technique in which researchers ask respondents to ‘list as many *X*s’ as they can, where *X* refers to some cultural domain” (Gravlee et al., 2018, p. 120). In our study, researchers asked the first 10 participants to list all animals they could think of (Figure 2). The remaining 9 participants were asked to list all the negative and positive elements they could identify in their kitchen, bathroom, or workspace. The free-list question changed because the researchers wanted to ‘test’ CDA online and then explore a type of question more closely related to their future study.

Some of the participants expressed that they were not able to think of any answers, which led facilitators to develop appropriate prompts, for example, asking participant to think about animals starting with different letters of the alphabet. Another strategy was to ask, ‘are there any animals similar to those already on your list?’

**Figure 3.**
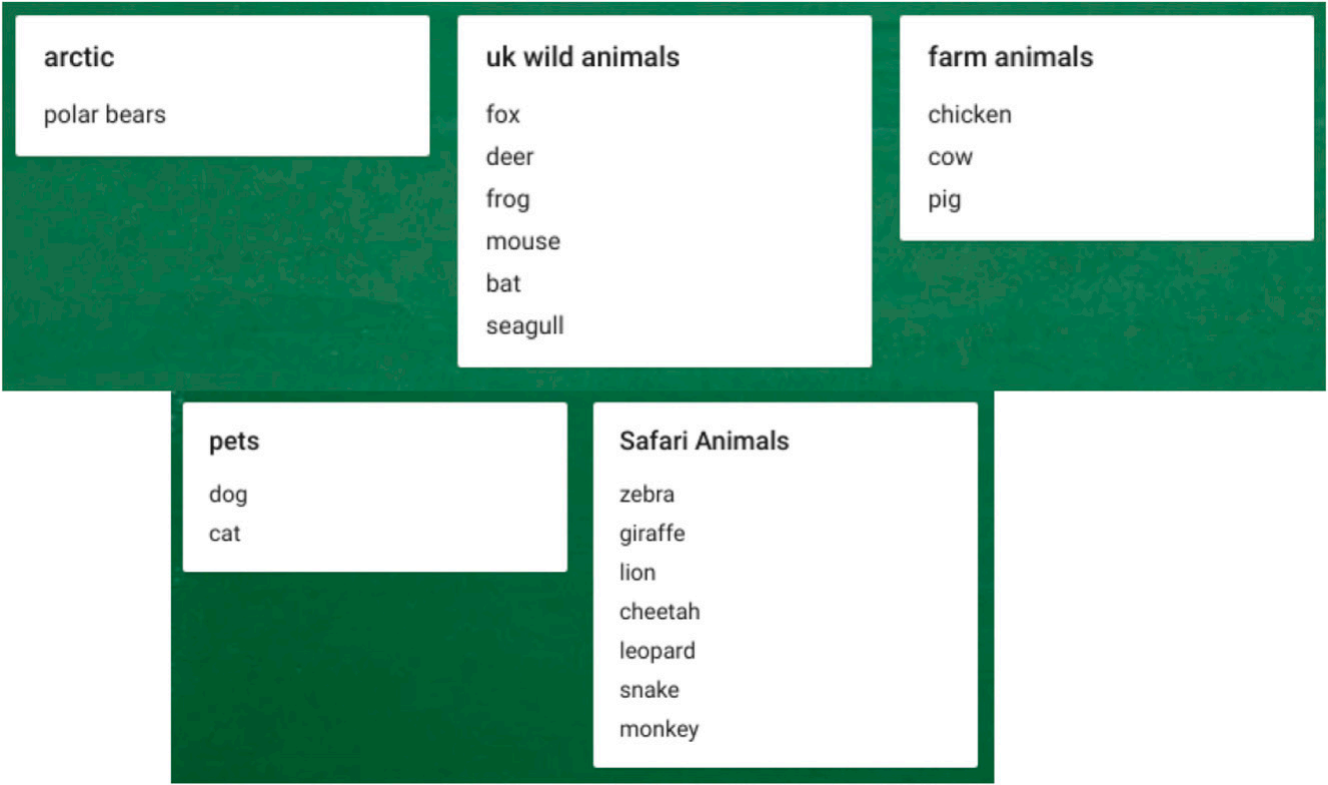
Example of a participant’s pile sort on Padlet®.

Following the free listing exercise, participants were asked to sort their answers into piles according to things they thought went together, known as pile sorts. The pile sort task is aimed at eliciting “judgements of similarity among items in a cultural domain” from respondents while also eliciting “the attributes that people use to distinguish among the items” (Borgatti, 1998, p. 12). Pile sorts help categorise items mentioned during the free listing exercise into piles based on how similar participants perceive them to be. For example, one participant created five animal groups (Figure 3). Pile sorts present two key benefits: firstly, informants might find it enjoyable, and, secondly, they can help effortlessly evaluate resemblances among very large numbers of items (Boster, 1994, p. 1). After grouping the words, we asked participants to describe the different categories they created. Finally, we exported the lists to a PDF and saved them in the secure database.

**Figure 4.**
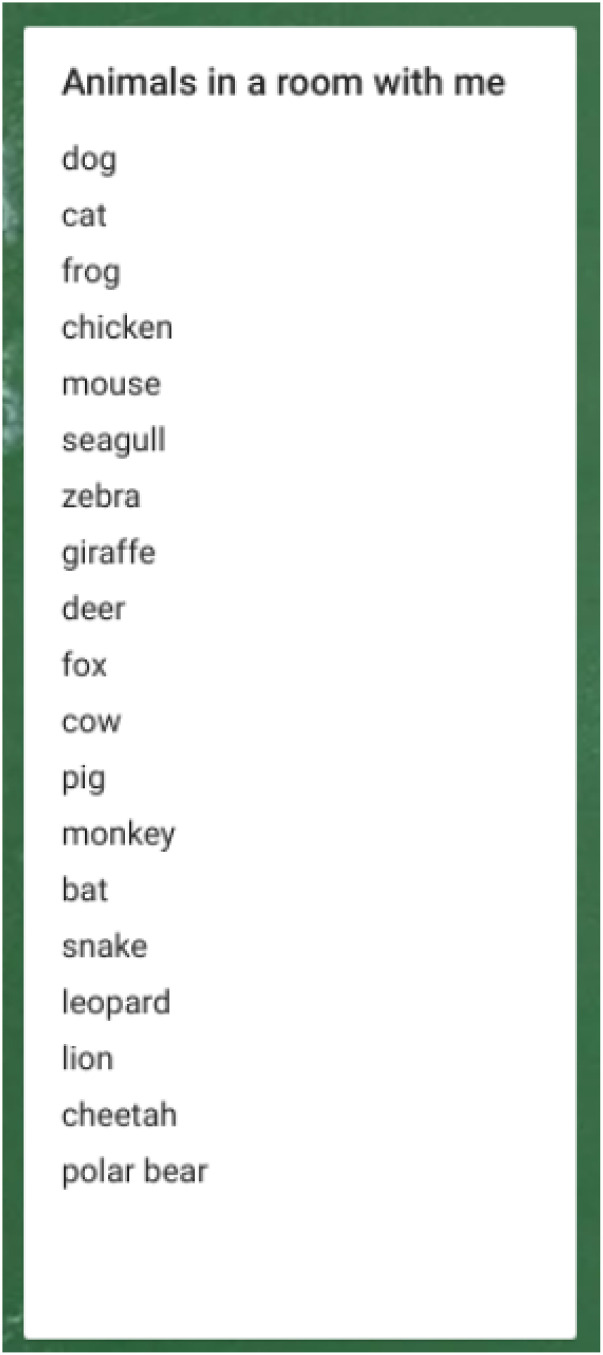
Example of a participant’s rank order on Padlet®.

The third task participants were asked to do was the step known as ‘rank order’, to rank the groups they created according to the animals they would feel most comfortable with in a room to the least (Figure 4). After ranking their answers, participants were asked to describe the rationale for their rank ordering.

Rank ordering is useful to identify what the most significant categories are for participants once they have put items into a pile. For example, significance could be related to benefits (what the most beneficial outcome is from a specific domain) or disadvantages (what the most negative element is). During the ranking exercise, researchers can also ask participants to rank the piles they have created from the most impactful to the least. This helps identify how participants perceive specific cultural domains, as well as compare their opinions by analysing differences and similarities between them.

### Phase 4: Data Analysis and Conclusion of the UAS

Once the first 10 sessions were completed, the research team evaluated the quality of the data and decided to include reflective questions at the end to better gain feedback on the overall research process. The sessions were useful to obtain opinions from participants, who provided advice on, among other things, the formatting of Padlet® (e.g., background, font, colours), accessibility, and support needs. This helped refine the questions asked and move the focus from the previous cultural domain of animals to asking participants to list the unmet needs they identified in either their kitchen, bathroom, or workspace better reflecting the variable of ‘unmet needs’ in the future study. When analysing the data gathered, the research team found that ‘unmet need’ was a difficult topic for participants to articulate, particularly when comparing this to listing the positive and negative elements about those spaces. We also realised that ‘unmet need’ was something that could only be realised over time; you do not know what you are missing until that need has been met or a certain level of reflection has been done. Reflecting on the study for which our UAS was conducted, we considered that, when conducting online CDA sessions with people living with HIV, participants might only be able to list what needs are lacking after some time. This indicates that an analysis based on listing positive and negative factors could better direct us to any unmet needs rather than asking directly about them.

## Participants’ Experiences

Participants identified issues with the Padlet® software, which, despite being described by some as “user friendly”, also presented challenges. One participant described that there were “too many items appearing on top of each other” (Participant 5). This was caused by a functionality in the software that, upon using the plus button instead of double clicking on the canvas, would create new post-it notes that hid the previous one. For the same participant, “it would have been easier to type the list within one tile [during the free listing exercise], as I could have cut and paste the items in different groups” (Participant 5). Participant 3 found the tasks easy to understand, but the high number of entries for the free listing technique and the functionality of Padlet® with a touch screen made “moving things around difficult”. We jointly opted for creating new post-its for each group and deleting the single entries to make the process easier. Frustration with the functionality was echoed by Participant 19 who said it was “difficult to control…moving the boxes…” but, “once you get used to it, it should be alright.” They felt it was “nice to have someone there to talk through it” and eventually felt “it was simple.”

A second issue had to do with feeling pressure about time, which made some participants feel like they had to complete the tasks quickly. Some expressed a lack of clarity about when to stop listing elements. One participant recommended the researchers rephrase the instructions by stating, “please let us know when you get stuck, otherwise we will let you know when to stop” (Participant 10). This insight led us to emphasise from the beginning of each session that there was no time limit, and that they could take as much, or as little time as required. 3 out of 19 participants also raised worries regarding spelling, which could have led some to not write specific terms should they feel shy about misspelling the words.

A third concern for a minority of participants related to the management of the audio recordings emerging from the sessions. This led us to realise that our consent forms had not included an explanation about the location where the recording would be stored securely and password protected, for how long, and who would be accessing them.^1^

An additional challenge related to the bad audio quality that some participants experienced, which led, together with the absence of a video screen showing the face of the researcher in some of the sessions, to difficulties in understanding what researchers were expressing. Lastly, a few participants stressed how face-to-face approaches could have facilitated a smoother explanation of the process.

Focusing on benefits, most participants agreed that taking part online had made it easier to join the project. One noted that he probably would have not participated otherwise given the time it would have taken him to travel from home. Another highlighted how his participation had been an opportunity to learn, as he described himself as someone who was “not computer literate.” This demonstrates the impact that online methods can have on participants as an educational source.

Others highlighted how participating online made them feel more comfortable than doing so in person. This was mainly because they did not feel ‘watched’. As one explained, “I feel alone and like I can do it [the task] by myself” (Participant 10). Another said that participating online “manages people’s anxieties” (Participant 12) because it allows one to be in a safe space. For another, this was positive because, “I didn’t notice that you were watching me; I feel my decisions were more authentic as you were not watching me” (Participant 5). Furthermore, some considered that using their laptop made participation easier because tools such as keyboards emerged as natural visual prompts through the keys versus the interviewers’ oral prompts. As a participant said, “I had my hand over the W key and just typed wallaby” (Participant 8). Another considered that the auto-correct option made her more confident to list answers and lessened any hesitation she might have had when writing by hand. However, one participant felt the feeling of rapport was lost online and suggested a warm-up exercise to make participants feel comfortable and to “give people a chance to talk about what they want” (Participant 16).

## Challenges and Benefits

Having discussed the three main phases of our study and participants’ views regarding their experiences, this section explores the challenges and benefits we identified from the implementation of CDA online.

Two main issues must be considered prior to implementing this type of approach to CDA. Firstly, researchers must contemplate whether our methodology is useful to collect data to address the specific research questions raised by their study. In our case, CDA was a pertinent tool to gather a large amount of information in a short period of time, allowing us to elicit elements constituting cultural domains and exploring how such elements interact, are organised and configured. Those aiming to explore domains will find CDA useful to quickly identify and describe aspects (e.g., drug treatment practices, perceptions on diseases, etc.) relevant to particular groups of people. Secondly, researchers should assess whether conducting research online is relevant and beneficial to their participants. For example, when working with vulnerable populations, it might be useful to offer the option to participate from home rather than having to travel to an external location. This, however, would require a level of digital literacy and accessibility that might not be available to all participants.

Turning now to the specific challenges and benefits identified in our UAS, what follows explores our experiences as researchers to discuss lessons learnt and recommendations for future research.

## Lessons Learnt and Recommendations

Drawing upon our UAS, key issues that researchers should consider for similar projects includes paying attention to the aesthetics of Padlet®; user accessibility and digital literacy; participants’ settings; Internet connection; reading body language online; and ethical considerations. Firstly, focusing on the software’s aesthetics, our UAS revealed that Padlet® backgrounds led to diverse reactions among participants. Some participants mentioned that the colours and prints had made them feel relaxed while writing down their answers, while others considered that a different combination of colours would have been better or giving directions describing shapes and text rather than colour. For example, one participant who was colourblind could not see the pink circle to create a new box and advised us to describe it as “a pink circle with a plus sign” (Participant 12) so that it would be more accessible to all participants. Among older participants and those with vision difficulties due to age, colour blindness or cataracts, a specific colour combination or bigger font size worked better to write and read.

Awareness of the importance of providing participants with accessible digital platforms is not recent, having been discussed in previous studies (Pearson & Koppi, 2002; Mankoff et al., 2005; Cobb & Sharkey, 2007). Such literature has emphasised the need to identify participants’ abilities and skills from the beginning of the recruitment process to make the software and digital platforms employed as accessible as possible. In light of our experience, it is recommended that future researchers reflect on the level of accessibility posed by the chosen software and discuss this with their institutional TEL team, which might be called differently depending on the institution. This discussion should not only consider health aspects (e.g., colour blindness, cataracts, hearing issues) but also the digital literacy of participants to find channels to support them to use the technology. If the interface used proves too difficult for a participant, they could dictate the words for the researcher to type them. Additionally, to increase their familiarity with the software, researchers could share a link to Padlet® or the relevant platform before the meeting to familiarise themselves. This could be accompanied by a short guide developed by the researchers on how to use the software.

Another issue to consider is the setting where sessions take place, which can impact how participants communicate with researchers. Our participants’ diverse profiles and the consequences of living in the midst of the COVID-19 pandemic led to changes in domestic life that affected their participation in the UAS. For those taking care of children at home, this meant that their participation could be disrupted. This context requires more flexibility to adapt to the unexpected through proactive strategies. Researchers could advise participants to use a separate room with no other people around to avoid being heard or having the voices of others in the recording. However, this might be difficult or impossible for those who do not have other rooms apart from those they share, or for participants with caring duties. Other daily events, such as a doorbell ringing, impacted our research when participants had to answer the door and returned being distracted from the task. This should be considered by the researchers to summarise what was being done before the interruption and ensure a smooth transition.

An additional issue to consider is how poor Internet connections or bad phone signal might impact communication with participants and, ultimately, data collection. These were issues we dealt with during our UAS, when Internet connectivity problems would impede hearing the participants’ voices. Those using their phone or tablet to join the sessions faced additional problems and required support from the researcher. For example, for Participant 11 the Padlet® screen disappeared making them remark “I now slightly regret doing [writing] so many [answers]. It’s so annoying!”. Another participant who participated on their phone was not able to move the tiles initially and with some difficulty managed but remarked, “I think my finger is too big!” (Participant 16). Additionally, using the phone means that receiving calls may affect what participants see on the screen and get distracted having to decline incoming calls. These issues must be considered in relation to the participants’ socioeconomic background, which may impact the level of Internet bandwidth and phone signal they have access to. Should they not have access to the technology required to participate, alternative options could be provided through vouchers to use the Internet or phone calls.

Complementing the themes mentioned above, a lesson learnt had to do with the difficulties to read body language. This was especially difficult because participants and researchers were looking at the Padlet® screen while listening to each other’s voices rather than seeing each other on the screen. While looking at the shared Padlet® screen, we often relied on the sounds that the participants made (e.g., groaning, talking to themselves) to discern what they were doing and if and when to probe. At times, it was difficult to ascertain whether the participants completely comprehended instructions without being able to fully see their reactions. This was even more complex when the participants kept their cameras turned off. Communication should always be central to overcome potential obstacles. Asking questions works better than telling participants they are being watched by the researcher, as our own experience indicates. In fact, for a couple of participants, being told that they were being observed on camera led to nervousness rather than comfort. Alternatively, if conducting the sessions individually it is possible to video record gallery view in Zoom for Education®, Microsoft Teams®, and Skype for Business®; however, ethical approval will need to be obtained beforehand. Alternatively, the sessions could be conducted with two researchers where one conducts the CDA session while the other uses screen view to monitor participants and note down non-verbal communication.

Lastly, ethical issues are a central component to consider when conducting digital research. As Trevisan and Reilly explain, online participants “should be protected from any additional harm that might arise from the use of their data in academic research through two processes, namely obtaining informed consent from the participant prior to the use of the data and the anonymization of data sets” (2014, p. 1137). Considering this, a key consideration from the beginning of our study was to provide participants with as much information as possible so that they could understand the study objectives and the consequences of participating. This was done through a participant information sheet and a consent form that participants were asked to fill before the sessions, when they were again reminded about the objectives and verbal consent was obtained.

Despite the similarities with traditional ethical issues, we came across a number of specific aspects that should be considered when doing research online, whatever the shape it takes (e.g., on the phone, laptop, tablet, etc.). For example, the fact that most participants joined our sessions from their homes, where they live their private lives, is an issue to consider when compared to interviews taking place in an academic office, or a public space. In addition to this, sharing the domestic space with other individuals such as housemates and family members led us to emphasise that participants should find a private space where they could participate by themselves. This study aimed to test CDA as a digital method, and we did not discuss any sensitive issues, however this must be considered for studies where participants discuss sensitive information that might be overheard by other people to ensure confidentiality and safety. Complementing the significance of evaluating the potential impact of the participants’ and researchers’ locations to ensure anonymity and confidentiality, using digital tools require considering risks associated with the potential leakage of data that could lead to losing confidentiality (Sparks et al., 2016, p. 41).

**Figure 5.**
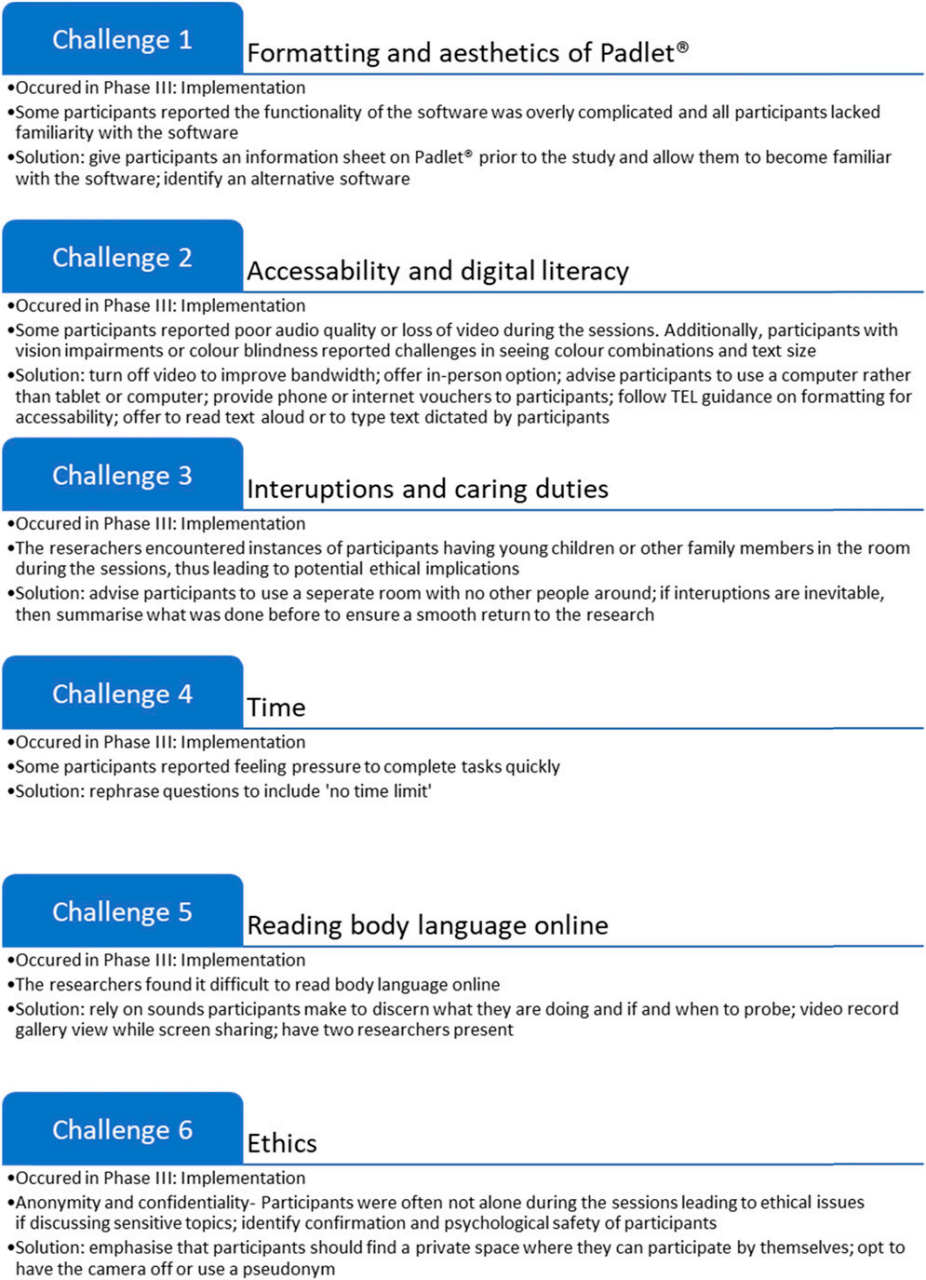
Challenges and solutions.

An additional ethical issue to consider with online research is anonymity. In this UAS staying on camera was not a study requirement because our subsequent HIV study provided participants with several options for creating anonymity, which we identified after consultation with a patient representative living with HIV. For example, they could opt to use a pseudonym during online sessions, and have their camera off. This is especially useful in small settings such as Brighton, where we conducted our study, since participants might know each other. Therefore, by giving them the option to use a pseudonym and/or to turn off their cameras, they could maintain anonymity. We wanted to offer the same options in the UAS to mimic the potential conditions of the HIV study; however, a majority of UAS participants kept their cameras on and nobody used a pseudonym despite one participant noting that using “a fake name might make them feel more comfortable” (Participant 13). Nevertheless, we recognise the importance of identify confirmation to group dynamics and psychological safety. Researchers should consider potential ethical issues that may emerge in their studies by considering our strategies for creating anonymity in recordings. We suggest researchers take a participatory approach to the study design and include representatives from the community or population under study, much like the use of patient representatives in our HIV study. Figure 5 below summarises the key challenges we faced and provides practical solutions that researchers could consider.

## Summary and Conclusion

This article has explored the process followed to conduct CDA online, while also presenting the challenges and benefits of doing so with the expectation that it can be valuable for other researchers. As noted, we conducted 19 CDA interviews online involving three main techniques: free listing, pile sorts, and rank ordering, all of which were completed successfully. Participants in the study had diverse backgrounds in terms of age, gender, and digital literacy, which helped us identify specific requirements that researching online could involve against more familiar in-person methods.

Our UAS illustrated the feasibility of conducting CDA online and from a distance. One of the main strengths of the UAS is that we tested the method as a standalone tool rather than assessing it within an ongoing research study. This allowed us to understand the method more deeply and to gather feedback for improvement so we could consider how it might be used in different research contexts. The primary contribution of this UAS lies in its provision of practical solutions to challenges that could be replicated by future research studies. Additionally, it is the only known published study that uses CDA online and from a distance during the COVID-19 pandemic. It is also one of the only known published studies that uses multiple CDA techniques (free listing, pile sort, and rank ordering) online and only the third published study that implements CDA online. It builds on our growing knowledge on the ways in which the method and its techniques can be deployed and adapted to different research contexts and situations.

Our UAS has pointed to a number of benefits that emerged from our approach. For participants, taking part online contributed to strengthening their digital skills, reduced travelling times by being able to join the sessions from home, increased their feeling of privacy, and facilitated reflecting on potential answers through prompts brought by the hardware used (such as their keyboards). Despite these positive outcomes, conducting CDA online also brought several challenges from which we learnt as data was collected. These included issues with Padlet® arising from the lack of familiarity with the software, user accessibility and digital literacy, poor Internet connection, difficulties to read the participants’ body language, specific device-related complications (when using a tablet or a phone), and distractions emerging from the home setting (e.g., doorbells, children) that interfered with the sessions. Identifying these challenges prior to starting research projects similar to the one we conducted, and formulating a clear response plan, can be appropriate solutions to overcome them.

When considering the implementation of research projects online, researchers must contemplate traditional ethical issues such as informed consent, ‘do no harm’ principles, anonymity, confidentiality, and data storage safety. Additionally, the blurred boundaries between public and private spaces in online research must be assessed to take steps to protect the participants’ safety. This includes physical safety and psychological safety. In the case of this study physical safety primarily refers to adhering to COVID-19 safety recommendations while psychological safety is much more difficult to assess, particularly in the online space. The research experience can be particularly impactful for individuals who may be marginalised, isolated, discriminated, or vulnerable. It is important for participants to know their views will be respected. We suggest following Williamson and Burns (2014) guidelines for participant psychological safety, which includes (1) being mindful of power imbalances between researcher and participant, (2) sensitively setting out the researcher and participant role including clear boundaries and safeguarding, and (3) informing participants regarding which circumstances and triggers they might benefit from follow-up work with other professionals.

In the midst of the COVID-19 crisis, researching from a distance and online provides us with opportunities to continue collecting data despite the restrictions that the pandemic has brought to our lives. Considering the lack of scholarship on digital CDA, it is our hope that future research continues to test the myriad of techniques that this methodology offers and to identify further benefits and challenges.

## Note

1. Our consent form only stated the sentence, “I agree to allowing the interview to be audio-recorded and for screen grabs/photographs of the computer screen to be taken”.
